# Medical instability in typical and atypical adolescent anorexia nervosa: a systematic review and meta-analysis

**DOI:** 10.1186/s40337-023-00779-y

**Published:** 2023-04-06

**Authors:** Cliona Brennan, Sarah Illingworth, Erica Cini, Dee Bhakta

**Affiliations:** 1 South London and Maudsley NHS Trust, Maudsley Hospital, De Crespigny Park, London, SE5 8AZ UK; 2 London Metropolitan University, 166-220 Holloway Road, London, N7 8DB UK; 3 East London NHS Foundation Trust, Emmanuel Miller Centre, 11 Gill Street, London, E14 8HQ UK; 4 Institute of Psychiatry, Psychology & Neuroscience, Kings College London, De Crespigny Park, London, SE5 8AB UK; 5 University College London, London, UK

**Keywords:** Anorexia nervosa, Atypical anorexia nervosa, Adolescent eating disorders

## Abstract

This review investigates the relationship between weight and risk of medical instability (specifically bradycardia, hypotension, hypothermia, and hypophosphatemia) in adolescents with typical and atypical anorexia nervosa. Atypical anorexia nervosa, listed as an example under the DSM-5 category of Other Specified Feeding and Eating Disorders (OSFED), describes patients who are not clinically underweight but otherwise meet criteria for anorexia nervosa. There is a lack of empirical evidence exploring medical complications in adolescents presenting with atypical anorexia nervosa. The small number of studies that do exist in this area indicate that medical instability exists across a range of weights, with weight loss being associated with increased medical risk, independent of underweight. The aim of this review was to collate and analyse results from available studies and identify indicators of medical risk in these two groups of adolescents with restrictive eating disorders. Studies were identified by systematic electronic search of medical databases, including PubMed and EMBASE. All studies investigated the relationship between weight and medical instability and included adolescents diagnosed with anorexia nervosa or atypical anorexia nervosa. One randomised controlled trial, five cohort studies and three chart reviews were included, with a total sample size of 2331 participants. Between 29 and 42% of participants presented with medical instability requiring hospitalisation, in the absence of underweight. Underweight adolescents were significantly more likely to have lower blood pressures (p < 0.0001) and bradycardia was significantly associated with greater weight loss (p < 0.05). There were no statistically significant associations found between degree of underweight and heart rate, temperature, or rate of weight loss (p = 0.31, p = 0.46 and p = 0.16, respectively). Adolescents that were less than 70% median body mass index were significantly more likely to have hypophosphatemia (p < 0.05). The findings of this review support the hypothesis that medical instability can occur across a range of weights in adolescent eating disorders, with rapid weight loss being an important indicator of increasing medical risk. Results were limited by the small number of existing studies that contained data for statistical analysis. Rapid weight loss should be considered as an important indicator of medical instability in adolescents presenting with both typical and atypical anorexia nervosa.

## Abstract (summary)

This review compares physical health markers of underweight and healthy weight in young people with restrictive eating disorders. Previous studies on this topic have found that young people with an eating disorder may become physically unwell regardless of their body weight. The aim of this review was to bring together the results from these studies to establish if the presence of underweight is necessary to become physically unwell. Studies within this review included a total of 2331 adolescent male and females diagnosed with an eating disorder. Roughly one third of young people with an eating disorder were found to be physically unwell and admitted to hospital but were not underweight, and this was similar to the amount of young people that were underweight and needed a hospital admission. Although some markers of physical health were worse in the underweight group, adolescents who were less underweight were also likely to become physically unwell. Overall, the review found that young people can become physically unwell regardless of whether they are clinically underweight or not, but that more research is needed on this topic.

## Background

Advancements in research in eating disorders have highlighted the importance of early identification of the illness and management of associated risks [1–3]. Degree of underweight as a definitive factor of illness severity has been challenged [4], whilst rapid weight loss, in the absence of underweight, has been identified as a key factor leading to medical instability and hospital admission in this patient group [5]. Further research in the area of weight and illness severity has been recommended by authors in the field, to improve assessment and management of atypical eating disorders [6]. The aim of this review was to explore the relationship between weight (% median BMI, absolute weight, speed and magnitude of weight loss) and specific markers of medical instability in adolescents presenting with typical and atypical anorexia nervosa.

## Introduction

### Diagnosis of restrictive eating disorders

Historically, in the 4th edition of the DSM, AN diagnosis was limited to those below a BMI of 17.5 kg/m^2^ in adults. In children and adolescents, percentage median BMI and percentage IBW are two widely used expressions of degree of underweight in adolescent eating disorders research [7]. However, there is currently no consensus on the best method to calculate IBW and different methods have been reported through the literature [8]. AN diagnosis in those under 18 years of age, was limited to those considered to be underweight, with a median BMI of < 85% being listed as an example of this in the DSM-IV [9]. This led to an assumption that specific weights were necessary to meet full criteria for illness. DSM-5 removed such examples and supported the use of clinical judgement in view of the overall presentation in diagnoses.

Amenorrhea was included within these diagnostic criteria [9]. These rigid criteria resulted in cases frequently being classed as subthreshold for diagnosis, despite presenting with clear symptoms requiring treatment, and were then categorised as EDNOS (Eating Disorders Not Otherwise Specified), as discussed further below. EDNOS was most frequently diagnosed in children and adolescents, as they typically presented with symptoms that did not meet full criteria for AN [10]. EDNOS encompasses a broad range of subthreshold disorders, and includes restrictive, binge-purge and non-underweight ED presentations. This heterogeneous diagnosis was problematic, with prognosis unknown, and treatment needs being unclear, thus unable to be targeted effectively [11].

In the updated DSM-5, diagnostic criteria for AN assessed severity based on both body mass and weight loss, rather than degree of underweight alone, and in the absence (or presence) of amenorrhea [12]. The presence of underweight is now based on clinical judgement, in the context of the overall presentation [12]. Nearly one third of patients formerly diagnosed with EDNOS are estimated to now meet criteria for AN according to the DSM-5 [13]. Improved specificity in classification aids earlier identification and improved management of the illness [1].

Patients who are not clinically underweight, but have lost significant amounts of weight, and otherwise resemble all characteristics of AN are now diagnosed with Other Specified Feeding or Eating Disorder (OSFED) (Table 1) [12, 14]. Atypical anorexia nervosa (AAN), a term used clinically to describe this patient group, is listed as an example of a presentation within OSFED. AAN remains a relatively new presentation, with a limited but quickly expanding evidence base related to its management and outcomes [4, 5, 15]. For the purpose of this review, the term AAN will be used to describe this group of non-underweight patients presenting with significant weight loss.

**Table 1:** DSM-5 Diagnostic criteria of anorexia nervosa and OSFED—Example 1. Atypical anorexia nervosa [12]

Anorexia nervosa	OSFED -Example 1. Atypical anorexia nervosa
A Restriction of energy intake relative to requirements leading to a significantly low body weight in the context of age, sex, developmental trajectory, and physical health B Intense fear of gaining weight or becoming fat, even though underweight C Disturbance in the way in which one's body weight or shape is experienced, undue influence of body weight or shape on self-evaluation, or denial of the seriousness of the current low body weight	All criteria are met, except despite significant weight loss, the individual’s weight is within or above the normal range

### Medical complications in AN

AN has, historically, had the highest mortality rate amongst all psychiatric conditions [16], with most deaths occurring between the ages of 16–29 years [17]. It is the third most common chronic illness among adolescent females [17, 18]. Restrictive eating, malnutrition and chronic low weight, all characteristic of AN, result in disturbances of the brain, reproductive, cardiovascular, gastrointestinal and skeletal systems [18–20].

Risk factors for serious illness or death are well defined in adolescent AN [21]. Key risk parameters are identified in the MEED national eating disorders guidance report (Table 2), alongside guidance on the recommended management. Medical consequences of AN in adolescence are described across many studies [1, 18, 22]. Significant bradycardia and low blood pressure, in particular, are common in AN, with these complications reported in both community and acute settings [23].

**Table 2:** Summary of MEED risk assessment framework of medical parameters [24]

Risk Domain	Parameter	High risk	High concern	Moderate risk
Body mass	%mBMI	< 70%	70–80%	> 80%
	Weight loss	1 kg/week	500–999 g/week	< 500 g/week
Cardiovascular health	Heart rate	< 40 bpm	40–50 bpm	> 50 bpm
	Blood Pressure	Standing systolic BP below 0.4th centile for age	Standing systolic BP below 0.4th centile for age	Normal standing systolic BP for age
	ECG	> 460 female, > 450 male	> 460 female, > 450 male	< 460 female, < 450 male
Hypothermia	Temperature	< 35.5	< 36	> 36
Biochemical abnormalities	Phosphate	Low	Low	Normal

Bradycardia is defined by a heart rate below 60 bpm, and is considered as significant bradycardia once heart rate falls below 50 bpm [24]. This complication has been shown to quickly resolve with nutritional rehabilitation and weight gain [25]. Degree of underweight and speed and magnitude of weight loss precede bradycardia, with research supporting weight loss, even in the absence of underweight, as a significant predictor [5]. Assalone and colleagues concluded in their study in 2021, that ‘recent weight loss is probably the most important determinant of severe bradycardia in adolescents with AN’ [26]. Similarly, cardiovascular complications such as hypotension and ECG abnormalities are widely reported in the literature in adolescent AN, and across a range of body weights [4, 27].

Hypophosphatemia, a hallmark feature of the refeeding syndrome, can occur during nutritional rehabilitation in adolescent AN [28]. Although prevalence of the syndrome across all ages in patients with AN is low (6–22%), it is associated with high morbidity and mortality [29]. Highest physical risk has been attributed to adolescents below 70% median BMI, presenting with rapid weight loss and severe energy restriction [30–32].

### Medical complications in AAN

Research and evidence relating to medical complications of AAN is limited [33]. Available literature relating to this subset of patients indicates that physical and psychological morbidity is similar to AN [15]. Adolescents with AAN frequently require acute hospital admission to treat medical complications related to weight loss and malnutrition, despite the absence of severe underweight [5].

A recent review, by Freizinger and colleagues, exploring assessment and treatment in adolescent AAN, in comparison to AN concluded that malnutrition related to rapid or significant weight loss, in the absence of underweight, poses similar risks to health as typical presentations of AN [34]. Similarly, a recent Delphi study exploring research priorities in AAN, identified medical complications arising from the disorder as a high priority [35].

### Current review

There is a lack of empirical evidence exploring medical complications in atypical anorexia nervosa presentations. The small number of studies and one existing systematic review in this area indicate that medical instability is similar across underweight and non-underweight adolescents [36]. This review draws together the evidence relating to weight parameters and risk of medical instability in typical and atypical anorexia nervosa.

## Methods

### Search strategy

**Fig. 1:**
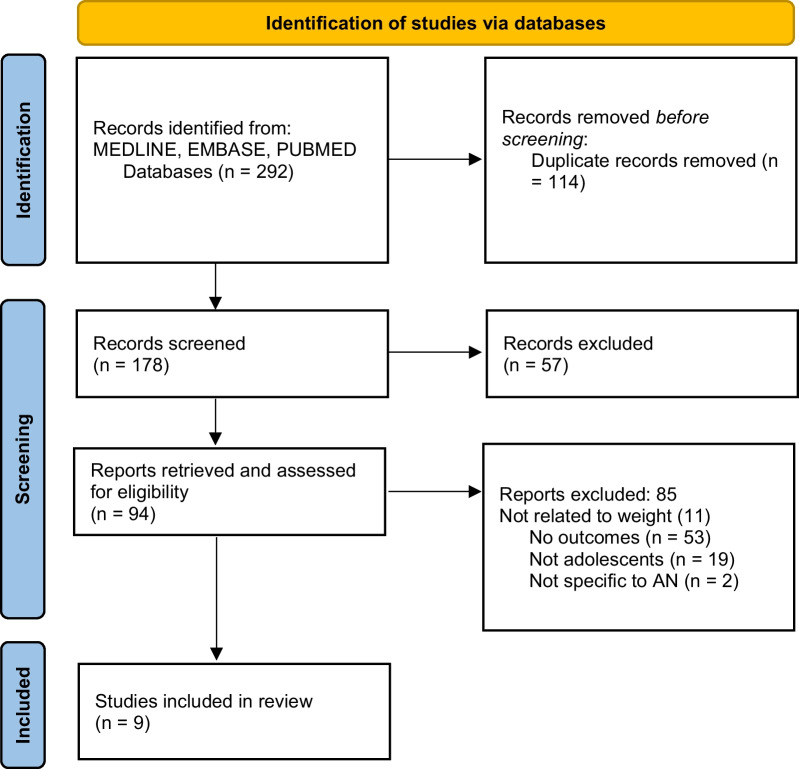
Selection of studies for this systematic review of the literature

Retrospective data from published studies related to weight and physical risk in young people with typical and atypical anorexia nervosa was systematically reviewed. Relevant studies were identified through electronic searches of databases including PubMed, MEDLINE and EMBASE, completed in September 2022, using key search words (anorexia nervosa, atypical anorexia nervosa, medical instability, bradycardia, hypotension, hypothermia, and hypophosphatemia). The process of selection and inclusion of studies in this review is displayed in Fig. 1. Two reviewers worked independently on study selection and agreed upon studies that met inclusion criteria for the review.

Studies were included if they involved research on children and adolescents, between 10 and 18 years old, presenting with AN or AAN, and investigated outcomes related to weight and acute markers of medical instability. Markers of weight included degree of underweight (expressed as %mBMI and %ideal body weight), speed and magnitude of weight loss (in kilograms). Medical instability outcomes recorded were heart rate, blood pressure, temperature, rate of weight loss and serum phosphorous. Studies were excluded if they were not in the English language, did not involve young people with AN, were not in peer reviewed journals or did not include markers of weight and outcomes relating to medical instability. References of included articles were screened for eligibility and included in the review if appropriate.

Two reviewers worked independently to extract data from studies selected for inclusion in the review. Corresponding authors of studies with missing data required for the meta-analysis were contacted. Studies were excluded from statistical analysis where missing data were not supplied; results of these studies were included in the narrative review. Data were recorded on a Microsoft Excel spreadsheet. Data extracted from studies included first authors name, year of publication, study design, sample size (including breakdown by diagnosis), each weight marker (degree of underweight and rate and magnitude of weight loss, and each outcome relating to medical instability (i.e., HR, BP, temperature, rate of weight loss and serum phosphorous).

Meta-analysis was carried out to compare medical instability outcomes between adolescents with AN and AAN. For this reason, only studies comparing two independent groups that contained data on medical instability outcomes could be included for statistical analysis. Groups were classified by their body mass; the AN group were classified by mBMI < 85%, and the AAN group as having mBMI > 85%.

Data were analysed using Review Manager version 5.4. The Mantel–Haenszel statistical model was used to compare means and standard deviations of the outcome measures (weight loss, HR, temperature, and BP, serum phosphorous) between the AN and AAN groups. Heterogeneity of the data were measured by chi-square. Significant heterogeneity was determined by a p value of less than 0.1 for the chi squared test for heterogeneity [37]. A random effects model was used for the full data set to account for significant heterogeneity. Sensitivity and specificity study outcomes were plotted onto a forest plot with the 95% confidence interval (CI). Statistical significance was determined by a p value of less than 0.05.

## Results

### Search results

An initial search of databases yielded 292 studies. Following the removal of 114 duplicates, the titles, and abstracts of the remaining 178 articles were screened. Ninety-four articles were identified for full paper review and were assessed for eligibility, of these a further 85 articles were excluded. Reasons for exclusion were a lack of relevant outcomes on medical instability (n = 53), subjects were not children or adolescents (n = 19), study did not include weight [11], and study was not specific to AN or AAN (n = 2).

The characteristics of the nine included articles are displayed in Table 3. All nine articles were based on adolescent subjects presenting with AN and AAN. All articles included outcomes related to weight and medical instability (HR, BP, temperature, and serum phosphorous). Three articles were included in statistical analysis, the remaining six articles were excluded as they did not include defined groups whose outcomes could be statistically compared.

**Table 3:** Characteristics of nine studies included in the review

Author	Population	Analysis	Results	Conclusions
Garber et al. [5]	12 to 24-year-olds with AN (n = 66) and AAN (n = 50)	Randomised controlled trial. AN and AAN groups were statistically compared using t test. Associations between weight history variables (total weight loss, rate of weight loss and duration of weight loss) and indicators of illness severity on admission were examined (HR and serum phosphorus)	Groups did not differ by weight history or admission HR. Independent of admission weight, lower HR (p = 0.01) was associated with faster loss; lower serum phosphorus was associated with a greater amount (p = 0.04) and longer duration (p = 0.001)	Greater rate, duration and amount of weight loss was associated with increased illness severity, regardless of current weight
Meirer et al. [2]	10 to 17-years-olds with AN. 2 cohorts (2004, 2014) 2 groups, premorbid overweight history (n = 34 and n = 16 in 2004) and premorbid normal weight history (n = 72 and n = 50 in 2014)	Single centre retrospective cohort study. Proportion of AN patients with a history of excess weight over a 10-year period examined. Premorbid normal and overweight history groups statistically compared in terms of medical markers at admission (HR, BP, and duration of amenorrhea) using t-tests	Excess premorbid weight was similar in both cohorts (32% in 2004 versus 29.5% in 2014). The historically overweight subgroup had a lower HR at intake (64.77 versus 69.75, p = 0.03). The total decrease in BMI greater in premorbid overweight group (7 BMI points versus 3.8, p = 0.0001)	Greater rate and amount of weight loss was associated with higher premorbid weight. Screening of AN should not be limited to the underweight and number of AAN cases increasing
Sawyer et al. [15]	13 to 17-year-olds with AAN (n = 42) and AN (n = 118)	Cohort study. Medical markers (HR, BP, temperature, and weight) and psychological markers of adolescents AAN and AN were compared using independent t-tests for continuous variables and χ2 for categorical variables	AAN lost more weight (17.6 kg vs 11.0 kg) over a longer period (13.3 vs 10.2 months) than AN. No significant difference in bradycardia rates (24% vs. 33%;) BP (43% vs. 38%). No difference found in frequency of psychiatric comorbidities (38% vs 45%)	Many adolescents with AAN present with medical instability, despite being healthy weight. Physical and psychological morbidity similar across AN and AAN, with similar proportions requiring hospitalisation and rates of AAN increasing
Whitelaw et al. [4]	12 to 19-year-olds with AN (n = 118) and AAN (n = 53)	Retrospective and prospective cohort studies comparing total and recent weight loss with admission weight as predictors of physical (hypophosphatemia, bradycardia, hypothermia, and hypotension) and psychological complications	Greater total weight loss (p = 0.002) and greater recent weight loss (p = 0.006), but not admission weight, were associated with a lower HR. Greater total weight loss (p = 0.003) and greater recent weight loss (p = 0.02) were associated with bradycardia	Number of AAN cases increasing over years. Weight loss was a stronger predictor than degree of underweight of physical complications leading to hospitalisation. Greater attention to weight loss as a measure of starvation is recommended
Whitelaw et al. [38]	12 to 19-year-olds with AN (n = 73) and EDNOS (n = 26)	6-year retrospective cohort study comparing HR, BP, and biochemistry of AN and EDNOS adolescents using linear regression for continuous variables, or nonparametric rank sum test for non-normal data	Proportion of EDNOS diagnosis increased from 8 to 47% over 6-year period. Hypophosphatemia developed in 41% of AN and in 39% of EDNOS patients. Lowest HR in AN was 45 bpm compared with 47 in EDNOS	Huge increase in the proportion of healthy weight adolescents hospitalised. EDNOS patients experienced a similar degree of medical instability as AN
Hudson et al. [1]	5 to 13-year-olds with AN (n = 76), BN (n = 3), EDNOS (n = 83), OSFED (n = 46)	Prospective surveillance study. T-tests and Mann–Whitney U tests were used to compare BMI, weight loss, HR, BP, and temperature between diagnoses	35% of cases had medical instability at presentation (60% bradycardia, 54% hypotension, 34% dehydration, 26% hypothermia). 52% of cases required admission at diagnosis (73% to a paediatric ward). 41% of cases had medical instability in the absence of underweight	Anthropological indices alone are poor markers for medical instability, clinical assessment is essential. Doctors providing care for children have central role in both the recognition and management of early onset EDs
Ornstein et al. [29]	8 to 22-year-olds with AN (n = 66)	Retrospective chart review. 3 groups (phosphorous < 2.5 mg/dl, < 3.0 mg/dl, and > 3.0 mg/dl) created. Age, weight, % IBW, and phosphorous were compared among groups. Pearson correlation coefficient was used to analyse the relationship between phosphorus and % IBW	Patients who developed moderate hypophosphatemia were significantly more malnourished than those who did not (p = 0.02). Phosphorus nadirs were directly proportional to % IBW (p = 0.01)	Those patients most severely malnourished are at the greatest risk for developing moderate hypophosphatemia
Peebles et al. [3]	8 to 19-year-olds with AN (n = 330), BN (n = 162), and EDNOS (n = 818)	Retrospective chart review. HR, BP, temperature and QTc compared between diagnoses using t-tests and × 2	EDNOS patients had the greatest and fastest weight lost (p = 0.001 and p = 0.005) and 28.7% compared with 38.5% AN patients had bradycardia. AN had highest rate of bradycardia (p = 0.01) and hypotension (p < 0.001) and lowest phosphorous levels (p < 0.005)	Medical complications can occur EDNOS and AN. Medical severity of patients with EDNOS intermediate to that of patients with AN
Whitelaw et al. [38]	12 to 18-year-olds with AN (n = 29)	Retrospective chart review study. Logistic regression used to identify associations between %IBW and phosphorous	%IBW at admission was significantly associated with the subsequent development of hypophosphatemia (p = 0.007)	Significant correlation between %IBW and incidence of hypophosphatemia

*N* sample size,* AN* anorexia nervosa,* BN* bulimia nervosa,* EDNOS* eating disorder not otherwise specified,* AAN* atypical anorexia nervosa,* RCT* randomised controlled trial,* HR*   heart rate,* BP*  blood pressure,* IBW*  ideal body weight,* QTc*  electrocardiogram measurement

All nine studies investigated the relationship between weight and medical instability in adolescent anorexia. Three main themes were identified across the articles: degree of underweight and medical instability, weight loss and medical instability, and degree of underweight and hypophosphatemia (in the context of refeeding syndrome risk).

All studies included adolescent participants, and these were mostly female. All nine studies included participants with AN. Four of the nine studies included patients with AN and AAN (diagnosed with EDNOS or OSFED), and two of the nine studies included patients with AN, EDNOS/OSFED and BN. Sample sizes ranged from 29 participants to 1310. Study designs included one randomised controlled trial, five cohort studies and three chart reviews.

### Degree of underweight and medical instability

Four studies focused on the relationship between degree of underweight and medical instability. The observational study by Hudson et al. [1] included participants diagnosed with AN, BN, EDNOS or OSFED (n = 208). Parameters of medical instability were available for 89% (n = 185) of all cases. Thirty five percent of these cases (n = 65) had medical instability upon presentation to hospital. Sixty percent had bradycardia (n = 39), 54% had hypotension (n = 35), 34% had dehydration (n = 22), and 26% had hypothermia (n = 17). Over half of cases (51%, n = 104) required hospital admission at diagnosis. Nearly half of cases (42%, n = 75) were medically unstable but were not underweight. Anthropological indices alone were deemed to be unreliable markers of medical stability in adolescents with EDs.

Sawyer et al. (2016) and Whitelaw et al. (2014) similarly found that markers of medical instability were not limited to underweight adolescents, with higher weight adolescents also frequently requiring hospital admission. In the study by Sawyer et al. (2016), over 40% of adolescents presenting with AAN required hospitalisation. Bradycardia, hypotension, and hypothermia rates were not significantly different between adolescent with AN or AAN. Adolescents with AAN lost more weight than those with AN (17.6 kg vs. 11.0 kg) over a longer period (13.3 vs. 10.2 months). The AAN group had a greater rate of weight loss than those with AN (1.3 kg/month vs. 1.1 kg/month). No significant difference in bradycardia rates (33% vs. 24%;) and BP (38% vs. 43%) were found between AAN and AN groups, nor were there any difference found in frequency of psychiatric comorbidities (45% vs. 38%).

In the retrospective study by Whitelaw et al. (2014), illness severity was compared between adolescents with AN and those presenting with AAN. Results showed that the proportion of adolescents presenting with AAN increased from 8 to 47% over the 6-year period. Hypophosphatemia developed in 41% of AN and in 39% of AAN patients and the lowest HR in AN was 45 bpm compared with 47 bpm in AAN. Authors concluded that adolescents with AAN present with similar physical complications as patients with AN, and that those with greater weight loss should receive close medical monitoring.

Peebles et al. (2010), found that adolescents presenting with AAN, who had lost > 25% of their premorbid body weight were physically compromised (i.e., presented with bradycardia, hypotension, and hypothermia) when compared with those who had not lost significant amounts of weight, independent of degree of underweight. The AAN group had a faster rate of weight loss when compared with the AN group (2.7% vs. 2.4% per month, p = 0.005). The AN group, compared with this AAN subset, had the highest rate of bradycardia (38.5% vs. 28.7%, p = 0.01) and hypotension (16.4% vs. 5.1%, p < 0.001) and highest incidence of hypophosphatemia (8.4% vs. 5.2%, p < 0.005). This study concluded that medical severity of patients with AAN was intermediate to that of underweight patients fulfilling all diagnostic criteria for AN.

### Weight loss and medical instability

Three studies looked at rate of weight loss in relation to illness severity and physical risk. In the randomised controlled trial by Garber and colleagues 2019, greater speed and magnitude of weight loss were significantly associated with lower HR and lower serum phosphorus, independent of weight. AN and AAN groups did not differ by weight history or HR. Independent of weight, lower HR was associated with faster rate of weight loss (p = 0.01); lower serum phosphorus was associated with a greater amount of weight loss (p = 0.04) over a longer duration of time (p = 0.001). Garber et al. (2019) concluded that weight loss should be considered as a key factor when assessing illness severity, regardless of degree of underweight [5].

In the study by Whitelaw et al. 2018, the relationship between weight loss and illness severity was explored. Greater total weight loss (p = 0.002) and greater recent weight loss (p = 0.006), but not admission weight, were associated with a lower HR. Greater total weight loss (p = 0.003) and greater recent weight loss (p = 0.02) were associated with bradycardia, defined as a heart rate lower than 60 bpm. The authors recommended that in adolescents presenting with AN or AAN, these two markers of weight loss were better predictors of medical instability than degree of underweight.

The cohort study conducted by Meirer and colleagues in 2019 investigated the relationship between premorbid overweight and weight history in adolescent AN, as well as the evolution of weight history over a 10-year period. Excess premorbid weight was similar in both the historically overweight and historically healthy weight cohorts (32% in 2004 versus 29.5% in 2014). The historically overweight subgroup had a lower HR at intake (64.77 versus 69.75, p = 0.03). The total decrease in BMI was greater in premorbid overweight group (seven BMI points versus 3.8, p = 0.0001). The authors recommended that with the number of AAN cases increasing, screening should not be limited to just the underweight.

### Degree of underweight and hypophosphatemia

The remaining two articles explored the relationship between degree of underweight and risk of refeeding syndrome complications, namely hypophosphatemia. Degree of underweight was expressed as %IBW rather than %mBMI in both studies. Ornstein and colleagues in 2003 found that hypophosphatemia was directly proportional to degree of underweight. Patients who developed moderate hypophosphatemia were significantly more malnourished than those who did not (p = 0.02). Phosphorus nadirs were directly proportional to degree of underweight (p = 0.01). Incidence of hypophosphatemia was greatest in adolescents that were less than 70% IBW. Extreme malnutrition and lower weight were concluded to put patients at greatest risk of hypophosphatemia.

The study by Whitelaw et al. (2010) had similar findings. Lower body mass at admission was significantly associated with development of hypophosphatemia (p = 0.007). Conclusions from this study, similar to that of Ornstein 2003, found that malnourished patients less than 70% IBW were at greatest risk of hypophosphatemia and that greater caution should be used in their management.

### Meta-analysis

**Fig. 2:**
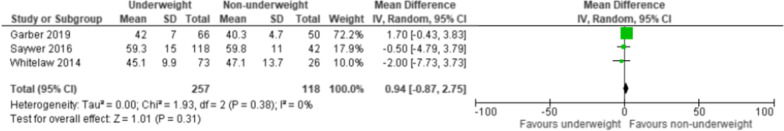
Forest plot showing association between degree of underweight and lower heart rate in adolescents with eating disorders

**Fig. 3:**
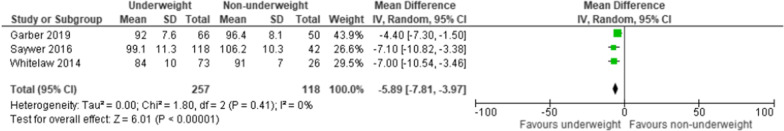
Forest plot showing association between degree of underweight and lower blood pressure in adolescents with eating disorders

**Fig. 4:**
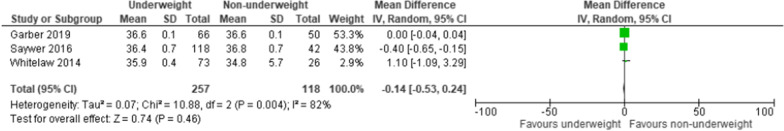
Forest plot showing association between degree of underweight and lower temperature in adolescents with eating disorders

**Fig. 5:**
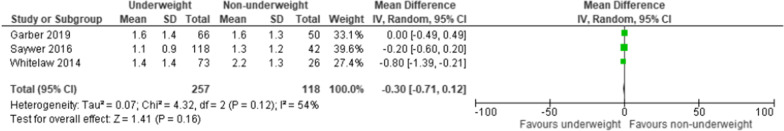
Forest plot showing association between degree of underweight and greater rate of weight loss in adolescents with eating disorders

Of the nine studies included in the narrative review, three of these contained data that could be extracted and statistically analysed. Authors of studies with missing data were contacted but failed to respond with required supplementary data for analysis. Studies included in meta-analysis were Garber et al. (2019), Sawyer et al. (2016), and Whitelaw et al. (2014). A meta-analysis was conducted where three or more studies contained data related to markers of medical instability that could be compared. Forest plots were generated and results of these are displayed in Figs. 2, 3, 4, 5.

In each of the four analyses, two independent groups (underweight AN and non-underweight AAN) were created and compared (Table 4). The underweight group included adolescents diagnosed with AN (n = 257), fulfilling full criteria for the disorder as specified by the DSM-5 including the presence of underweight. Median BMI ranged from 74.5 to 79%. The non-underweight group (n = 118) included adolescents who fulfilled partial criteria for AN but were not underweight, thus diagnosed with OSFED or EDNOS, and described as the AAN group in this review. Median BMI ranged from 93 to 106%.

**Table 4:** Characteristics of underweight (AN) and non-underweight (AAN) adolescents compared by meta-analysis

	n	HR (bpm)	Temperature (°C)	BP (mmHg)	mBM**I**	Phosphorous (mmol/L)
*AN (n = 257)*						
Garber et al. [5]	66	42 (7)	36.6 (0.1)	92 (7.6)	76.5% (5.9)	1.2 (0.2)
Whitelaw et al. [38]	73	45.1 (9.9)	35.9 (0.4)	84 (10)	74.5% (7.1)	1.2 (0.1)
Sawyer et al. [15]	118	59.3 (15)	36.4 (0.7)	99.1 (11.3)	79% (7.1)	n/a
*AAN (n = 118)*						
Garber et al. [5]	50	40.3 (4.7)	36.6 (0.1)	96.4 (8.1)	95.2% (9)	1.3 (0.2)
Whitelaw et al. [38]	26	47.1 (13.7)	34.8 (0.7)	91 (7)	93% (5.3)	1.2 (0.1)
Sawyer et al. [15]	42	59.8 (11)	36.8 (0.7)	106.2 (10.3)	106% (13.4)	n/a

Groups were comparable in terms of age and gender and differed by their percentage median BMI. Both groups included male and female adolescents, presenting with symptoms of AN. Heart rate, BP, temperature, and rate of weight loss at the time of initial assessment were statistically compared between groups. Serum phosphorus could not be compared as only two studies contained data related to this marker. Thus, the relationship between hypophosphatemia and degree of underweight could not be fully investigated.

Results identified a statistically significant relationship between degree of underweight and blood pressure. Underweight adolescents were more likely to have lower BP than those that were not underweight (p < 0.00001). There was no statistically significant association found between degree of underweight and any other markers investigated in the analysis, including HR, temperature, and rate of weight loss (p = 0.31, p = 0.46 and p = 0.16, respectively).

## Discussion

### Overview of results

This review aimed to determine firstly, the relationship between weight and medical instability in adolescents with typical and atypical anorexia nervosa, and secondly, if underweight adolescents with AN are at greater risk of medical instability than non-underweight adolescents with AAN.

Findings from the narrative review identified that medical instability occurs in a large proportion of adolescents presenting with AN and AAN, across a range of body weights. Results indicated that the speed and magnitude of weight loss perpetuate medical instability in both underweight and non-underweight groups. Low heart rate and blood pressure were the most likely symptoms of malnutrition to occur across both groups.

Only a small number of studies (n = 3) reported results that could be included in statistical analysis. Results from meta-analysis identified underweight adolescents with AN as having significantly lower BP than non-underweight adolescents presenting with AAN. All other markers of medical instability were similar across groups, with no significant differences identified.

### Medical instability in AN and AAN presentations

Evidence has shown that prevalence of AAN has increased over the past decade [4]. Despite presenting as clinically non-underweight, hospital admissions are often required in this group, with research showing similar rates to their underweight counterparts [2, 15, 39]. Considering this growing number of medically unstable, but non-underweight adolescents, comprehensive medical assessment of all adolescents presenting with restrictive eating disorders is recommended [1, 2].

Results from this study indicated that medical instability and complications related to starvation were not limited to underweight adolescents. Healthy weight adolescents were found to have a similar degree of medical instability as those classed as underweight, although the lower weight group did have significantly lower blood pressures. Bradycardia and hypotension were the most common presenting symptoms, and rates were similar across underweight AN and non-underweight AAN groups. Rates of bradycardia ranged from 29 to 60% and rates of hypotension ranged from 40 to 54%, across all diagnoses. Hospitalisation was also common across groups, with ~ 40% of non-underweight adolescents in studies reviewed requiring a medical admission. These results correlate with findings of other recent reviews that compare AN and AAN [11, 34].

Statistical analyses found that underweight adolescents with AN had significantly lower BP than non-underweight adolescents with AAN. However, these results were limited by the few studies containing sufficient data that could be analysed (three out of nine studies). Additionally, groups compared by meta-analysis did not differ significantly in rates of weight loss preceding assessment. Therefore, results should be interpreted cautiously, with underweight adolescents being at significantly higher risk of having lower BP than those that are not underweight, in the absence of significant differences in rates of weight loss. Results may differ in non-underweight adolescents that have lost significant amounts of weight. The severity of certain medical markers, such as BP, is likely worsened in adolescents who present with extremes of low weight in addition to rapid weight loss.

### Medical instability and weight loss

In adult AN, weight suppression (difference between weight at assessment and premorbid weight) has emerged as a marker of illness severity, independent of degree of underweight [5]. Weight suppression, in individuals presenting at normal weights, has been associated with medical instability (e.g., bradycardia) [4]. In the RCT conducted by Garber and colleagues in 2019, weight loss was identified as a key marker of malnutrition and determinant of hospitalisation, independent of other factors [5].

In our review, findings indicated that greater magnitude and speed of weight loss predicts physical complications requiring hospitalisation, independent of degree of underweight. In addition, adolescents of a higher pre-morbid weight were found to have greater weight loss than those of a lower weight [4, 5]. Results from the narrative part of this review support weight loss as a predictor of medical complications, in addition to degree of underweight. Adolescents presenting with greater total, duration, and rate of weight loss in addition to low weight are at greatest risk of bradycardia and hypotension. However, due to the lack of statistically significant difference in weight loss rates between underweight and non-underweight groups, meta-analysis could not be used to compare physical risk by weight loss rate.

### Hypophosphatemia and weight

All reviewed studies were in agreement that a positive relationship between the degree of malnutrition and hypophosphatemia on admission exists [40]. The degree of malnutrition could be used as an indicator for pending hypophosphatemia but is not a definitive risk factor, with both underweight and non-underweight adolescents with AN and AAN being at risk [31].

Hypophosphatemia was identified across both underweight and non-underweight groups. However, results from the narrative review identified adolescents < 70% mBMI as being at significantly greater risk of developing this symptom. Considering the findings of this review, paired with results from recent research studies, adolescents < 70%mBMI may be considered at greatest risk of developing hypophosphatemia during nutritional rehabilitation [29, 32]. Other factors, such as rapid weight loss, further increase this risk [5].

### Strengths

The key strength of this review stands in it being one of the few systematic reviews of this nature, looking specifically at evidence relating to weight and risk of medical instability in adolescents with typical and atypical anorexia nervosa. Although there have been multiple review papers that look at anorexia nervosa and physical health, none provide a clear analysis of the objective significance of weight parameters in assessing risk of physical deterioration.

This review brings together the limited evidence that is available on this topic. It highlights the need for further research studies investigating the factors associated with physical risk in AN and AAN, across underweight and non-underweight adolescents.

### Limitations

The results of this review are limited by the small number of studies that met inclusion criteria and that could be analysed. Many studies included in the narrative review lacked comparable data, meaning they were excluded from statistical analysis, limiting the power of the findings of the meta-analysis. Ethnic variation could not be considered in meta-analysis. This limits the accuracy of results due to cut-offs for thinness varying across ethnicities. Similarly, the high degree of heterogeneity across the sample limits the power of the results from this statistical analysis. Different methods of expressing degree of underweight were used across studies included in the narrative review, limiting the comparability of study results. However, studies included in the statistical analysis were consistent in their use of %mBMI to express degree of underweight, which minimises the effect this factor had on these results.

There is a growing body of evidence on adolescent eating disorders. However, high quality intervention studies are hugely lacking. This review identified many studies for inclusion, however, there was a lack comparable data that could be quantitively analysed.

## Conclusions

Current National guidance specifies the range of risks associated with starvation and stratifies this risk for each marker [24]. Greatest risk is attributed to those < 70% mBMI, as well as those presenting with greatest weight loss. This review draws together currently available evidence and indicates that greatest risk of medical instability occurs in adolescents who are most severely malnourished. This may be evidenced by their extremes of low body weight, their rapid weight loss, or a combination of these two factors.

Future work should focus on studies that can be compared to current evidence, to allow a more thorough analysis of data relating to weight parameters and their significance in typical and atypical adolescent anorexia nervosa. Further research is essential in this area, particularly in non-underweight adolescents with AAN to help clinicians understand medical risks arising in the absence of underweight.

## Data Availability

All data generated or analysed during this study are included in this published article.

## Abbreviations

ED: Eating disorder
AN: Anorexia nervosa
DSM: Diagnostic and statistical manual
BMI: Body mass index
%mBMI: Percentage median body mass index
KG: Kilogram
M^2^: Metres squared
W4H: Weight for height
IBW: Ideal body weight
EDNOS: Eating disorder not otherwise specified
AAN: Atypical anorexia nervosa
OSFED: Otherwise specified feeding or eating disorder
MEED: Managing emergencies in eating disorders
BPM: Beats per minute
ECG: Electrocardiogram
RS: Refeeding syndrome
CI: Confidence interval
mmHG: Millimetres of mercury
RCT: Randomised controlled trial
